# Discovering broad-spectrum inhibitors for SARS-CoV-2 variants: a cheminformatics and biophysical approach targeting the main protease

**DOI:** 10.3389/fphar.2025.1459581

**Published:** 2025-02-05

**Authors:** Safar M. Alqahtani

**Affiliations:** Department of Pharmaceutical Chemistry, College of Pharmacy, Prince Sattam Bin Abdulaziz University, Al Kharj, Saudi Arabia

**Keywords:** COVID-19, SARS-CoV-2, BBB-26580140, BDE-32007849, LAS-51378804, molecular dynamics simulations, water swap method

## Abstract

The COVID-19 pandemic caused by SARS-CoV-2 still lacks effective antiviral drugs. Therefore, a thorough receptor-based virtual screening study was conducted to screen different natural and synthetic drug libraries, such as the Asinex Antiviral, Seaweed Metabolite Database, Medicinal Fungi Secondary Metabolite and Therapeutics Library, and Comprehensive Marine Natural Products Database comprising 6,827, 1,191, 1,830, and 45,000 compounds, respectively, against the main protease enzyme of SARS-CoV-2. Accordingly, three drug molecules (BBB-26580140, BDE-32007849, and LAS-51378804) are highlighted as the best binding molecules to the main protease S1 pocket. The docking binding energy scores of BBB-26580140, BDE-32007849, and LAS-51378804 were −13.02, −13.0, and −12.56 kcal/mol, respectively. Compared to the control Z1741970824 molecule with a binding energy score of −11.59 kcal/mol, the lead structures identified herein showed robust hydrophilic and van der Waals interactions with the enzyme active site residues, such as His41 and Cys145, and achieved highly stable binding modes. The simulations showed a stable structure of the main protease enzyme with the shortlisted leads in the pocket, and the network of binding interactions remained intact during the simulations. The overall molecular mechanics with generalized Born and surface area solvation binding energies of the BBB-26580140, BDE-32007849, LAS-51378804, and control molecules are −53.02, −56.85, −55.44, and −48.91 kcal/mol, respectively. Similarly, the net molecular mechanics Poisson–Boltzmann surface area binding energies of BBB-26580140, BDE-32007849, LAS-51378804, and control are −53.6, −57.61, −54.41, and −50.09 kcal/mol, respectively. The binding entropy energies of these systems showed lower free energies, indicating their stable nature. Furthermore, the binding energies were revalidated using the water swap method that considers the role of the water molecules in bridging the ligands to the enzyme active site residues. The compounds also revealed good ADMET properties and followed all major rules of drug-likeness. Thus, these compounds are predicted as promising leads and can be subjected to further experimental studies for evaluation of their biological activities.

## 1 Introduction

Wuhan city in the Hubei province of China experienced an outbreak of pneumonia with unknown etiology around the end of December 2019. Up until 31 January 2020, the outbreak had grown significantly, infecting 9,720 people and resulting in the deaths of 213 people in China as well as 106 persons in 19 other countries (Carabelli et al., 2023). The coronavirus pandemic of 2019 first started in Wuhan city and was declared a pandemic by the World Health Organization (WHO) when the disease spread across the globe (Excler et al., 2021; Omolo et al., 2020). It was found to be caused by the highly contagious severe acute respiratory syndrome coronavirus 2 (SARS-CoV-2) microorganism (Viana et al., 2022). This virus is grouped under the Coronaviridae family and has an RNA genome with distinct crown-like spikes on the surface (Dai et al., 2020). As of 23 November 2024, this disease is responsible for 7,010,681 deaths resulting from 704,753,890 confirmed cases of infection in 231 countries (https://www.worldometers.info/coronavirus/). Although substantial efforts have been made in the last 2–3 years to better understand the biology of SARS-CoV-2, outbreaks of the disease are still being reported in many countries (Msemburi et al., 2023).

The COVID-19 pandemic was triggered by SARS-CoV-2. Since the coronavirus 3-chymotrypsin-like protease (3CLpro) regulates viral replication, it is considered as a key target and potential approach for the development of direct-acting rationally based antiviral drugs (Vandyck and Deval, 2021). Different mutants of the virus have been reported to be associated with the newer outbreaks (Khan et al., 2021). This high mutagenicity of the virus is attributable to its RNA genome, which acquires genetic adaptations to rapidly form new variants with different characteristics than the ancestral strain (Korber et al., 2020). The WHO has thus far reported five SARS-CoV-2 variants based on epidemiological data, namely the omicron (B.1.1.529), alpha (B.1.1.7), beta (B.1.351), gamma (P.1), and delta (B.1.617.2) types (Carabelli et al., 2023). The treatment options available for COVID-19 and recommended by WHO are as follows: baricitinib, sotrovimab, molnupirvir, remdesivir, sarilumab, and tocilizumab as IL-6 receptor blockers (Mousavi et al., 2022). Despite the availability of these drugs, COVID-19 remains an emergent disease for which development of new treatments is required. SARS-CoV-2 codes for at least four structural proteins that help with viral RNA synthesis, viral assembly process, and viral binding to the host cell receptors (Kim et al., 2020). The structural proteins include spike, membrane, envelope, nucleocapsid, and 16 non-structural proteins (Calligari et al., 2020). The main protein enzymes, also known as chymotrypsin-like and papain-like proteases, are the two proteases vital for coronaviral proteolytic processing of polyproteins (Narayanan et al., 2022). The main protease is the key enzyme of SARS-CoV-2 biology as well as functionality and is significant as a drug target (Agost-Beltrán et al., 2022). The main protease enzyme was found to be the target in about 154 research studies, including both *in silico* and experimental works, and several compounds have been reported against it. Natural compounds are rich sources for drug development owing to their strong bioactivities and structural variety, which have long been recognized as important criteria. For instance, research has shown that plant-derived alkaloids and flavonoids can effectively block important enzymes linked to diseases because of their distinct chemical structures (Chopra and Dhingra, 2021). As proven by the production of sulfonamide derivatives with increased inhibitory activities against bacterial enzymes, synthetic compounds have the advantage of customization for particular biological targets integrating specific strategies; a new study has shown how natural scaffolds and synthetic shifts work collaboratively to improve the therapeutic design efficacy and specificity (Dzobo, 2022). Several inhibitors have been proposed against the main protease enzyme of SARS-CoV-2. For example, PF-07304814, PF-07304814, and PF-07304814 are phase 1 trial inhibitors proposed by Pfizer. Ebselen and masitinib proposed by Sound Pharmaceuticals and AB Science, respectively, are currently under phase 2 trials (Pang et al., 2023).

The development of new drug molecules is a complicated, expensive, and lengthy process that takes approximately 2–4 years of preclinical efforts and up to 6 years of clinical studies (Maltezou and Papa, 2011; Shaker et al., 2021), with the total procedural costs often being as much as 500 million dollars. Conventional drug discovery entails three vital steps, namely discovery and development of targets or leads, preclinical research, and clinical development (Yu and MacKerell, 2017). Drug discovery also includes hit screening, applications of medicinal chemistry, and structural/biological activity optimizations to limit the side effects of the drugs (Gediya and Njar, 2009); these steps often involve the use of either classical or reverse pharmacology. Both of these technologies have their pros and cons. Computational drug discovery has been proven significant in the recent past for its utility in identifying and optimizing lead structures (Van Drie, 2007). Computational and biophysics techniques could reduce time and deliver hits that can directly be used in experimental work. The present computational study aims to identify novel inhibitors against the main protease enzyme of SARS-CoV-2 using different techniques through computational tools like UCSF Chimera, Discovery Studio, AMBER package, and SwissADME.

## 2 Materials and methods

**FIGURE 1:**
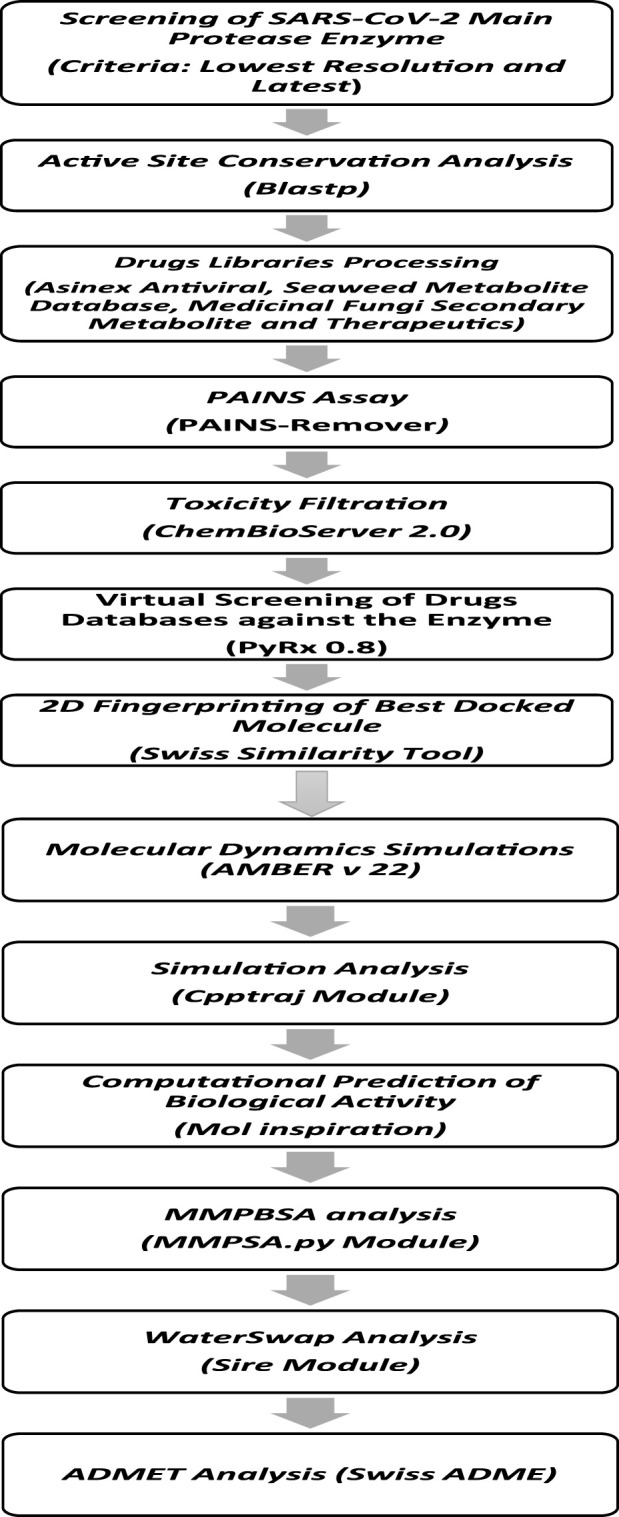
Comprehensive stepwise approach used to identify potential inhibitors against the main protease enzyme of SARS-CoV-2. First, the high-resolution structure of the enzyme was retrieved, followed by structural preparation and energy minimization. Then, several drug libraries were screened against the target enzyme with the aim of prioritizing potential non-toxic and non-PAINS inhibitors with stable binding conformations as well as interactions with the enzyme active pocket. Multiple postsimulation analyses were performed on the simulation trajectories to validate the docking findings.

The methodological steps of this study, starting from enzyme retrieval to receptor-based virtual screening and biophysics techniques, are presented in Figure 1.

### 2.1 Main protease enzyme crystal structure searching, retrieval, and processing

An initial exhaustive search was performed for the best resolution and latest structure deposited in the protein data bank (PDB) from the start of the COVID-19 pandemic up to 5 September 2023. This allowed identification of the crystal structure of the protease enzyme (PDB ID: 5RF3) in complex with the compound Z1741970824 (Douangamath et al., 2020). The structure was deposited to the PDB on 15 March 2020 and released on the same day. The structure was also determined by X-ray diffraction and is available at the best resolution of 1.50 Å. This structure is expressed in *Escherichia coli*, which was used as the expression system. These findings suggest the determination of a high-quality main protease structure. Next, the structure was imported into UCSF Chimera v1.17 (Kaliappan and Bombay, 2018), where it was first visualized for missing residues and structural errors. The missing hydrogen atoms and charges were added using “AddH” and “Add Charge” features in Chimera v1.17 (Kaliappan and Bombay, 2018). Energy minimization was then carried out to relax the enzyme structure, and the process was completed using two algorithms. The first algorithm was the steepest descent method, which was implemented for 5,000 cycles with a step size of 0.02 Å. The second algorithm used was the conjugate gradient method, which was also implemented for 5,000 steps. Both these algorithms ensured the removal of steric clashes present in the enzyme structure.

### 2.2 Active site conservation analysis

The active site conservation analysis was performed using the main protease enzyme sequence of SARS-CoV-2 from the given PDB ID. The sequence was imported into the Blastp tool of NCBI and searched against the PDB (Mahram and Herbordt, 2015). A multiple sequence alignment (MSA) was performed, and the conservation of two important catalytic residues (His41 and Cys145) at the S1 pocket of the enzyme was examined (Elsässer et al., 2017). A complete conservation was observed, illustrating the key mechanistic roles of these residues in enzyme catalysis.

### 2.3 Drug library selection, retrieval, and processing

A thorough literature search was conducted to determine the drug libraries that best meet the current research aim. The selection of libraries was based on the following criteria: chemical scaffold diversity, easy availability for experimental assays, most extracted data from natural sources, and not much explored in past studies. Accordingly, the Asinex antiviral library (Yadav et al., 2021), Seaweed Metabolite Database (Davis and Vasanthi, 2011), Medicinal Fungi Secondary Metabolite and Therapeutics Library (MFSMTL) (Vivek-Ananth et al., 2021), and Comprehensive Marine Natural Products Database (CMNPD) (Sheokand et al., 2024) were found, which have 6,827, 1,191, 1,830, and 45,000 compounds, respectively. The Asinex antiviral library was designed from the perspectives of safety profile and profound antiviral activity. The Seaweed Metabolite Database comprises compounds extracted from marine algae. The MFSMTL is a curated database of natural products and contains secondary metabolites extracted from diverse medicinal fungi. The CMNPD is rich in marine compounds extracted from bacteria, fungi, green algae, sponges, cnidarians, echinoderms, etc. After retrieval, the libraries were subjected to pan-assay interference compounds (PAINS) filtration to remove false positive compounds (Whitty, 2011). Thereafter, toxicity filtration was performed to remove toxic chemical scaffolds from the filtered PAINS libraries; this was accomplished using the ChemBioServer 2.0 server (Athanasiadis et al., 2012). Then, the filtered compounds were imported into PyRx 0.8 for energy minimization (Dallakyan and Olson, 2015), where the libraries were energy minimized using the MM2 force field (Halgren, 1996) and converted to .pdbqt format.

### 2.4 Site-directed virtual screening

Site-directed virtual screening of the selected drug libraries against the S1 pocket of the main protease enzyme was conducted in PyRx 0.8 (Dallakyan and Olson, 2015). One of the essential enzymes that convert the viral polyprotein into useful components required for viral replication is the SARS-CoV-2 major protease (Mpro). This enzyme is an important target for treatment as it effectively stops the viral replication cycle (Pang et al., 2023). The grid boxes were set around His41:ND1 (*x*-axis: 12.676 Å, *y*-axis: −4.526 Å, *z*-axis: 21.049 Å) and Cys145: N (*x*-axis: 6.416 Å, *y*-axis: −5.110 Å, *z*-axis: 16.812 Å) (Douangamath et al., 2020). The box size along each dimension was set to 30 Å. The number of binding conformations generated for each compound at the active pocket of the enzyme was 100. Then, iterations with binding energy scores ≥ −5.00 kcal/mol were discarded. A control molecule in the form of Z1741970824 was used to test and validate the docking protocol. This control molecule was extracted from the crystal structure and docked with the enzyme at the same position using PyRx 0.8, and the root mean-squared deviation (RMSD) value was calculated using UCSF Chimera v1.17 (Kaliappan and Bombay, 2018). It was observed that both the crystal and PyRx 0.8 docked conformations were similar, with an RMSD value of 0.15 Å. The best-docked solutions from virtual screening were filtered and used in Discovery Studio Client v2021 to thoroughly examine the chemical interactions (Biovia, 2017). The binding conformations of the selected compounds with the enzyme were studied using USCF Chimera v1.17 (Kaliappan and Bombay, 2018).

### 2.5 2D fingerprinting of the best-docked molecules

The 2D fingerprinting of the best-docked molecules was performed using the Swiss similarity online server (Zoete et al., 2016). The main objective here was to search for the derivatives of leads identified in the virtual screening process and to evaluate their binding affinities with the main protease enzyme.

### 2.6 Molecular dynamics simulations: systems preparation and production

Molecular dynamics simulation is a powerful biophysics tool that provides insights into complex dynamics over the simulation time (Ahmad et al., 2017; Rafi et al., 2020). The simulations were carried out using AMBER v22 (Case et al., 2022). The systems were preprocessed using the Antechamber program (Wang et al., 2001). The leap module was applied to add missing hydrogen atoms (Schafmeister et al., 1995). The systems were solvated into the OPC water model and counter ions were added subsequently to neutralize the charge on the complexes (Sengupta et al., 2021). To describe the receptors, the FF19SB force field was used. The compounds were treated using the general Amber force field 2 (GAFF2) (Tian et al., 2019; Vassetti et al., 2019), and the energy minimization was achieved using 500 iterations of the steepest descent and conjugate algorithms. During this process, the protein was fixed with a force constant of 250 kcal/mol/Å^2^. The systems were heated to 300 K under a constant volume (NVT) ensemble for 20 ps. At the same time, a weak constraint of 10 kcal/mol/Å^2^ was applied to the protein atoms. Then, ensemble equilibration of the systems was performed for approximately 1 ns under NPT. The production iterations comprised 100-ns runs with an average pressure of 1 atm using isotropic position scaling. Langevin dynamics was applied for temperature control in the presence of a collision frequency of 1 ps^−1^ (Izaguirre et al., 2001). The long-term non-bonded interactions were handled using the particle mesh Ewald method (Petersen, 1995). The hydrogen atoms involved in bonding were constrained by the SHAKE method (Kräutler et al., 2001). The simulation trajectories were determined using the CPPTRAJ script of AMBER for structural stability investigations (Roe and Cheatham, 2013). Lastly, XMGRACE v5.1 was used to obtain the plots (Turner, 2005).

### 2.7 Estimating binding free energies

Estimation of the binding free energies is vital in the current drug discovery pipeline because of the modest computational requirements and prediction accuracy. The binding free energies are considered to be more accurate than the docking calculations as the former is based on simulation trajectory frames and considered as dynamic conformations of the intermolecular binding; the latter is based only on one docked intermolecular conformations (Wang et al., 2019). The binding free energies were estimated using the molecular mechanics Poisson–Boltzmann surface area (MM-PBSA) approach in AMBER v22. The MM-PBSA was obtained using the AMBER MMPBSA.py module (Miller III et al., 2012). The initial .prmtop files for the complexes, receptors, and ligands were generated using the ante-MMPBSA.py module. The MM-PBSA is based on the principle of subtraction of the receptor and ligand energies from that of the complex (Genheden and Ryde, 2015). Mathematically, the MM-PBSA is given by

$${∆G}_{\text{net}\;\text{binding}\;\text{energy}}=G_{\text{complex}\;\text{binding}\;\text{energy}}-\left(G_{\text{protein}\;\text{binding}\;\text{energy}}+G_{\text{ligand}\;\text{binding}\;\text{energy}}\right).$$

Individually, the G terms above comprise the molecular mechanics energy, coupled temperature and entropic energy, and solvation free energy, as given by the following equation:

$$G=E_{\text{molecular}\;\text{mechanics}}-\left(\text{TS}\right)+\left(G_{\text{solvation}}\right).$$

The MM-PBSA energy equation was simulated on a total of 10,000 frames. The binding entropies for the complexes were obtained separately using normal mode analysis in AMBER (Genheden et al., 2012). Owing to the computationally intensive nature of the entropy energy calculations, only five frames were processed.

### 2.8 Water swap analysis

The water swap approach is considered to be a more robust and theoretically efficient method for predicting the absolute binding affinity of a guest ligand to a given macromolecule (Woods et al., 2014). Here, a protein ligand is swapped with an equal volume of water clusters present in the protein active pocket (Bergström and Larsson, 2018). This method entails constructing a λ-coordinate that allows the joining of a periodic box of water molecules with a periodic box of ligands. The energy change in the λ-coordinate is predicted by standard methods used in water swap (Ahmad et al., 2019) over a default of 1,000 iterations.

### 2.9 Prediction of ADMET properties

Predictions of the ADMET properties of the compounds are vital for selecting viable and effective molecules for *in vitro* and *in vivo* testing (Van De Waterbeemd and Gifford, 2003). Compound failure during clinical trials due to poor ADMET properties incur both financial and time costs to scientists (Jia et al., 2019). Therefore, it is preferable to select compounds that are predicted to possess favorable ADMET properties as these are more likely to clear clinical hurdles associated with their chemical attributes. The ADME properties of the compounds were determined using SwissADME (Daina et al., 2017), while their toxicities were predicted through the pkCSM server (Pires et al., 2015).

### 2.10 Computational prediction of the biological activity

The bioactivity scores of the selected compounds were evaluated using an online Mol inspiration server. This server predicts the bioactivity score of a given compound against the ion channels, kinases, proteases, G-protein-coupled receptors (GPCRs), and enzymes. The Mol inspiration server can be accessed using the following link: https://www.molinspiration.com/cgi/properties.

## 3 Results

### 3.1 Virtual screening for hit compounds

**FIGURE 2:**
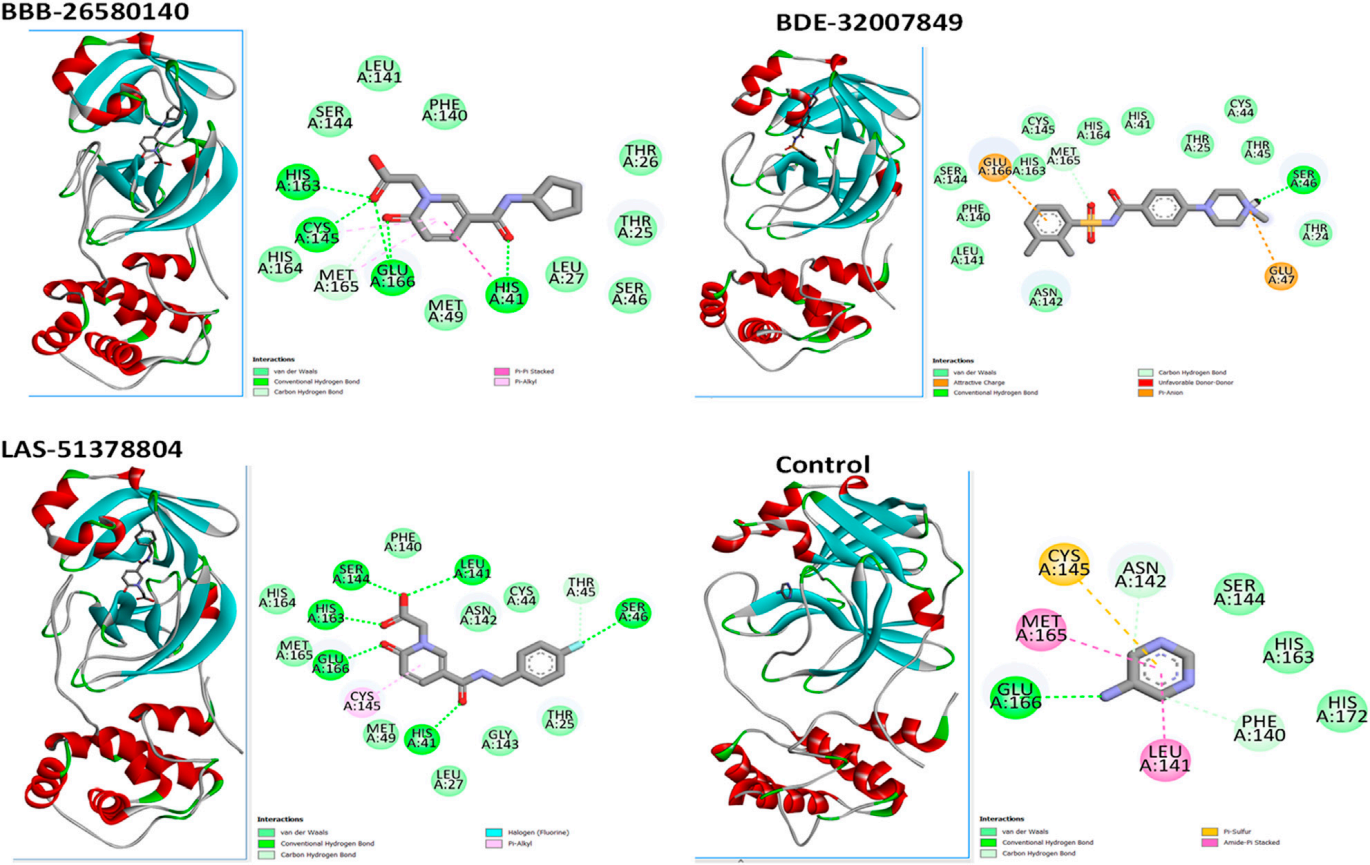
Binding modes and interactions of the leads with the S1 active pocket of the main protease enzyme of SARS-CoV-2. The different types of interactions are presented in the figure.

A structure-based virtual screening protocol was applied to screen compounds from the selected drug libraries. The non-PAINS and non-toxic compounds are only considered as discussed in the methods section. The screening identified three promising compounds from the libraries, namely BBB-26580140, BDE-32007849, and LAS-51378804, with binding energy scores of −13.02, −13.0, and −12.56 kcal/mol, respectively. These compounds showed better binding affinity scores than the control (Z1741970824; binding energy score: −11.59 kcal/mol). The lead structures also showed robust hydrophilic and hydrophobic interactions with the enzyme active site residues (His41 and Cys145) and achieved highly stable docked binding modes. The interaction patterns of the compounds were observed under broad and expanded conditions for the S1, S1′, S2, and S3 active pocket regions. All three compounds showed deep bindings at the active pocket and were found to have adjusted well inside the pocket. The chemical names of BBB-26580140, BDE-32007849, and LAS-51378804 are 1-(carboxymethyl)-5-(cyclopentylcarbamoyl)pyridin-1-ium-2-olate, 4-(4-(((3-chloro-2-methylphenyl)sulfonyl)carbamoyl)phenyl)-1-methylpiperazin-1-ium, and 1-(carboxymethyl)-5-((4-fluorobenzyl)carbamoyl)pyridin-1-ium-2-olate, respectively. The BBB-26580140 compound formed hydrogen bonds with His41, Cys145, His163, and Glu166 with interaction lengths of 1.56 Å, 1.84 Å, 1.64 Å, and 2.0 Å, respectively; the compound also showed hydrophobic interactions with Leu141, Ser144, Phe140, Thr26, Thr25, Ser46, Leu27, Met49, Met165, and His164. The BDE-32007849 compound produced a hydrogen bond with Ser46 at a bond length of 1.87 Å; this hydrogen bond was formed through the compound terminal benzene ring. The remainder of the compound structure (N-((3-chloro-2-methylphenyl)sulfonyl)benzamide) formed hydrophobic contacts with His41, Thr25, Cys44, Thr45, Thr24, Ser144, Leu141, Phe140, Asn142, Glu47, and Glu166, among others. The LAS-51378804 compound interacted majorly through its 1-(carboxymethyl)-5-(methylcarbamoyl)pyridin-1-ium-2-olate part and formed hydrogen bonds with Ser144, His163, Glu166, and His41 at bond lengths of 1.56 Å, 2.01 Å, 1.56 Å, and 1.42 Å, respectively. Fluorobenzene also formed a hydrogen bond with Ser46 at a bond length of 2.14 Å. The docked conformations and chemical bondings of the compounds and control are given in Figure 2. These results illustrate that the compounds are deeply and strongly bound to the main protease enzyme S1 pocket, resulting in highly stable binding conformations. Furthermore, each of the lead molecules was used in a similarity-based 2D search, and the best results against each lead were tabulated.

### 3.2 2D fingerprinting of the best-docked molecules

Two-dimensional fingerprinting analysis was performed against the drug-like molecules from the PDB, which allowed shortlisting of 15 compounds for BBB-26580140, six compounds for BDE-32007849, and 16 compounds for LAS-51378804. The prediction scores against each of the derivatives are given in Table 1. The compounds were redocked with the protease enzyme using the same protocols and parameters. None of the structurally similar compounds were found to have better binding than the parent structure.

**TABLE 1:** Structure-based compounds similar to those of the leads.

Lead/Similarity-based scaffolds	BBB-26580140	BDE-32007849	LAS-51378804
Parent structure	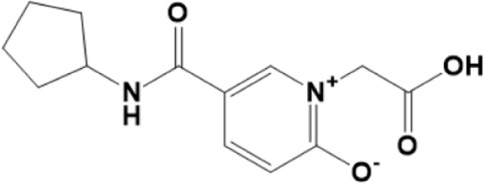	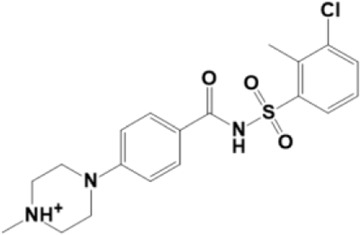	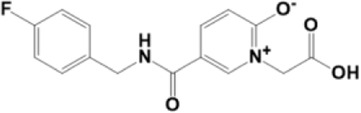
	DB06397, Nicaraven Score: 0.540 Docking score: −8.21 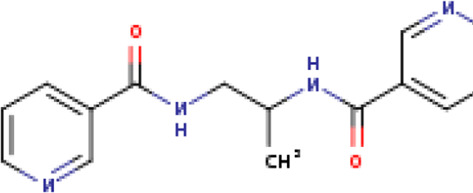	DB09355, Sulfabenzamide Score: 0.642 Docking score: −7.52 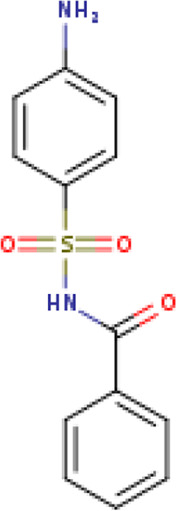	DB15450, PF-05105679 Score: 0.542 Docking score: −6.34 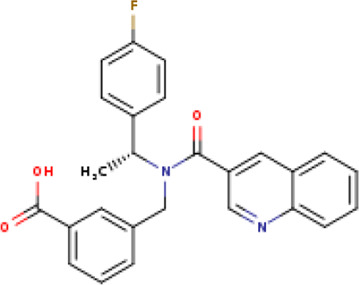
	DB12929, JNJ-39220675 Score: 0.536 Docking score: −9.62 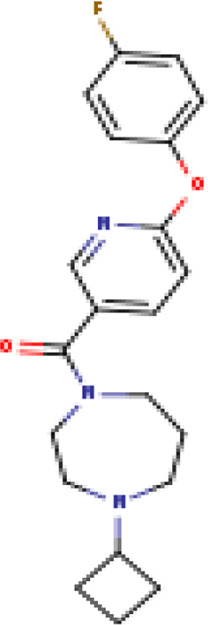	DB12418, Saccharin Score: 0.607 Docking score: −6.35 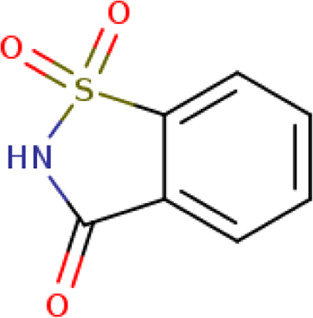	DB12929, JNJ-39220675 Score: 0.539 Docking score: −7.54 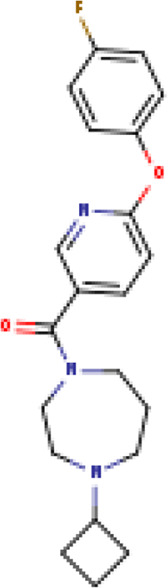
	DB13655, Nikethamide Score: 0.524 Docking score: −7.54 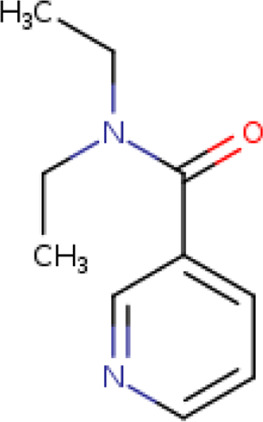	DB07115, N-(4-chlorobenzyl)-N-methylbenzene-1 Score: 0.575 Docking score: −5.32 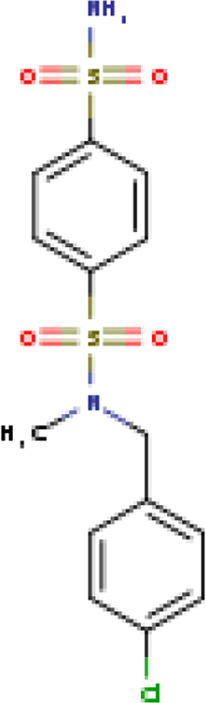	DB12585, Ondelopran Score: 0.535 Docking score: −9.64 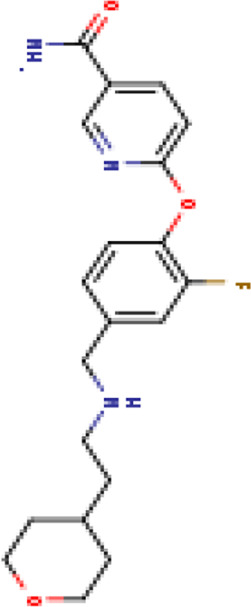
	DB12585, Ondelopran Score: 0.523 Docking score: −10.25 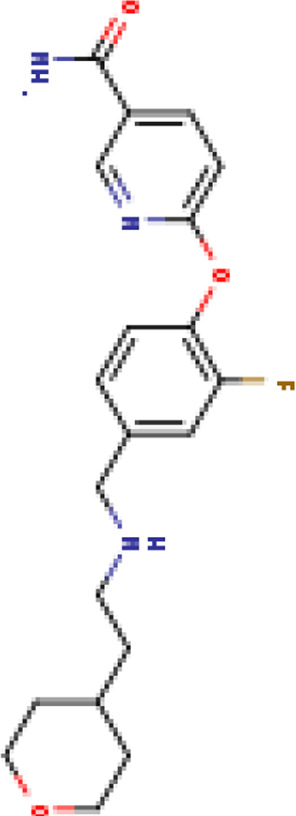	DB16040, DU125530 Score: 0.536 Docking score: −9.64 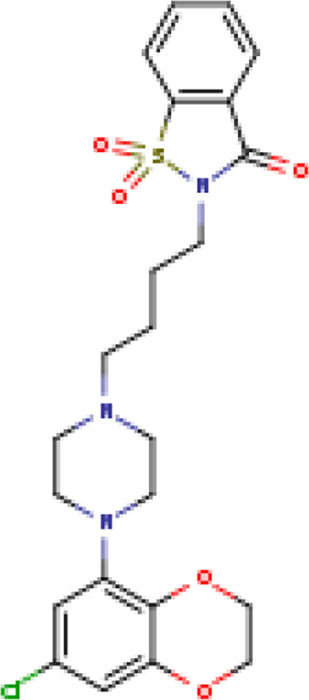	DB11366, Roquinimex Score: 0.527 Docking score: −6.98 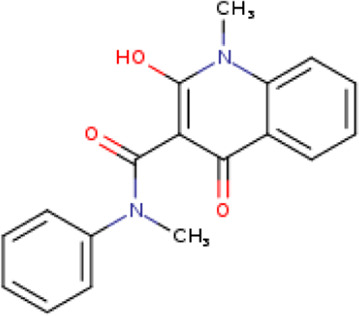
	DB04764, [4-(3-aminomethyl-phenyl)-piperidin-1-yl]-(5-phenethyl- pyridin-3-yl)-methanone Score: 0.522 Docking score: −6.54 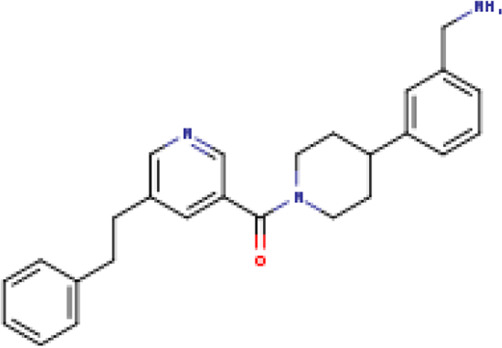	DB07476, n-[4-(aminosulfonyl)phenyl]-2-mercaptobenzamide Score: 0.484 Docking score: −7.84 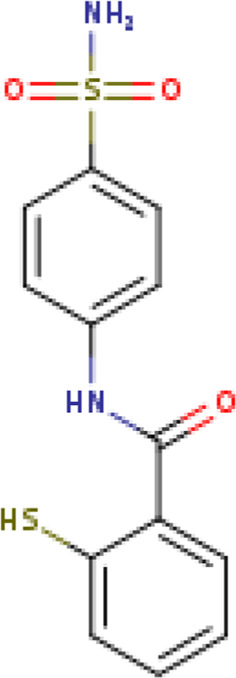	DB12562, Setipiprant Score: 0.509 Docking score: −4.45 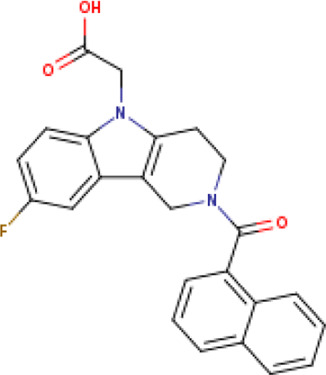
	DB11366, Roquinimex Score: 0.503 Docking score: −10.065 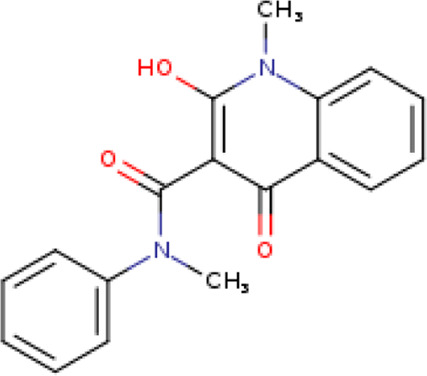	DB07049, (2R)-1-[(4-tert-butylphenyl)sulfonyl]-2-methyl-4-(4-nitrophenyl)piperazine Score: 0.482 Docking score: −9.65 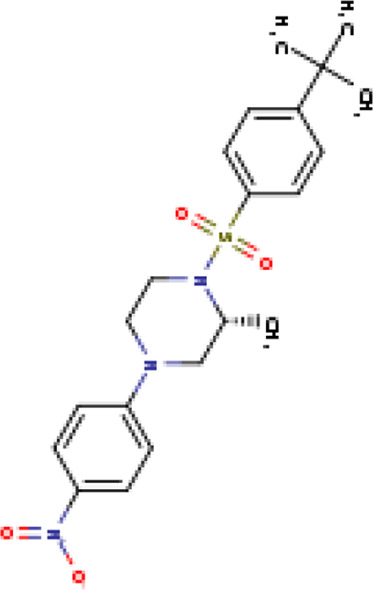	DB13327, Picotamide Score: 0.508 Docking score: −8.01 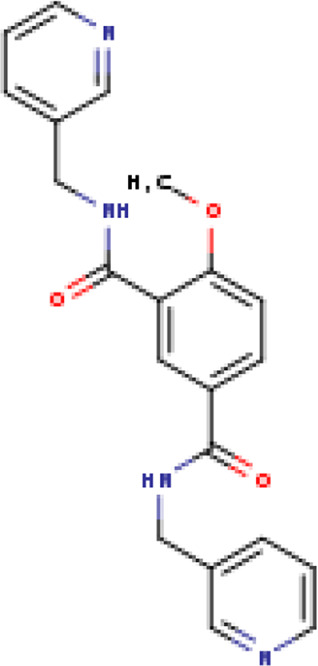
	DB14805, DCFPyL F-18 Score: 0.500 Docking score: −8.65 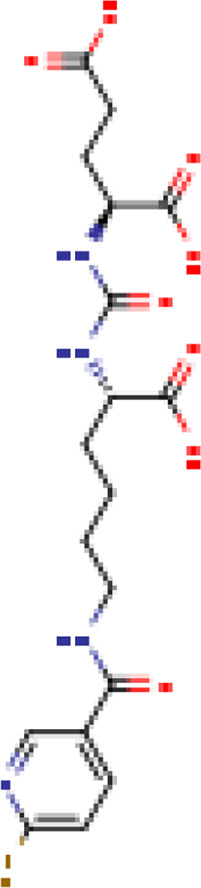	Not predicted	DB05861, Tasquinimod Score: 0.506 Docking score: −9.64 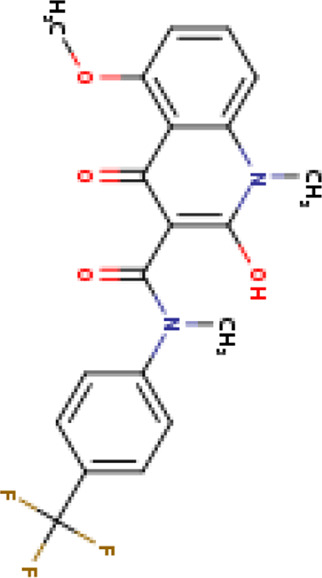
	DB13531, Nicofetamide Score: 0.492 Docking score: −8.21 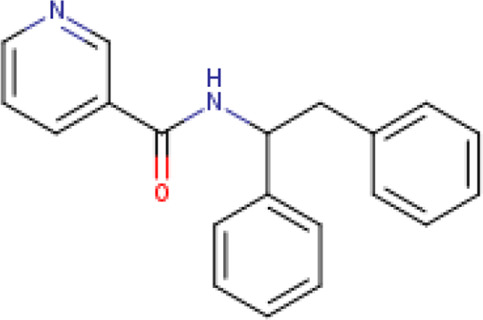	Not predicted	DB13655, Nikethamide Score: 0.500 Docking score: −6.58 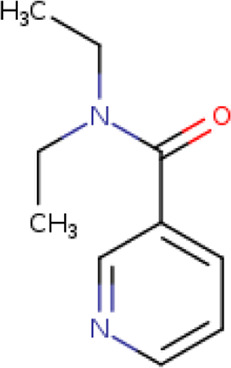
	DB13687, Niaprazine Score: 0.489 Docking score: −8.64 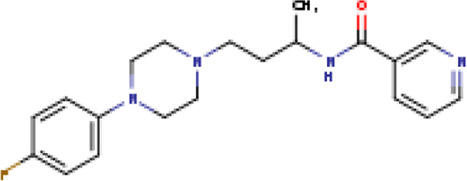	Not predicted	DB13531, Nicofetamide Score: 0.496 Docking score: −9.64 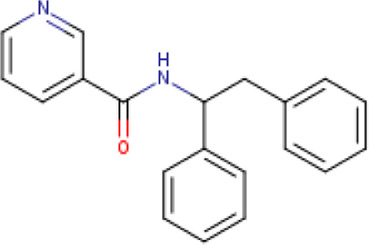
	DB04873, Piboserod Score: 0.488 Docking score: −8.54 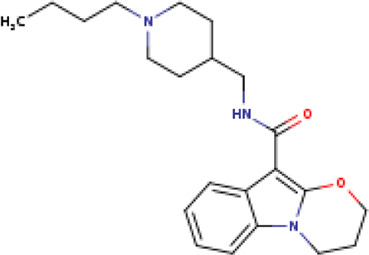	Not predicted	DB13687, Niaprazine Score: 0.493 Docking score: −8.64 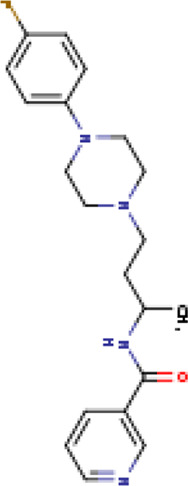

### 3.3 Molecular dynamics simulations

**FIGURE 3:**
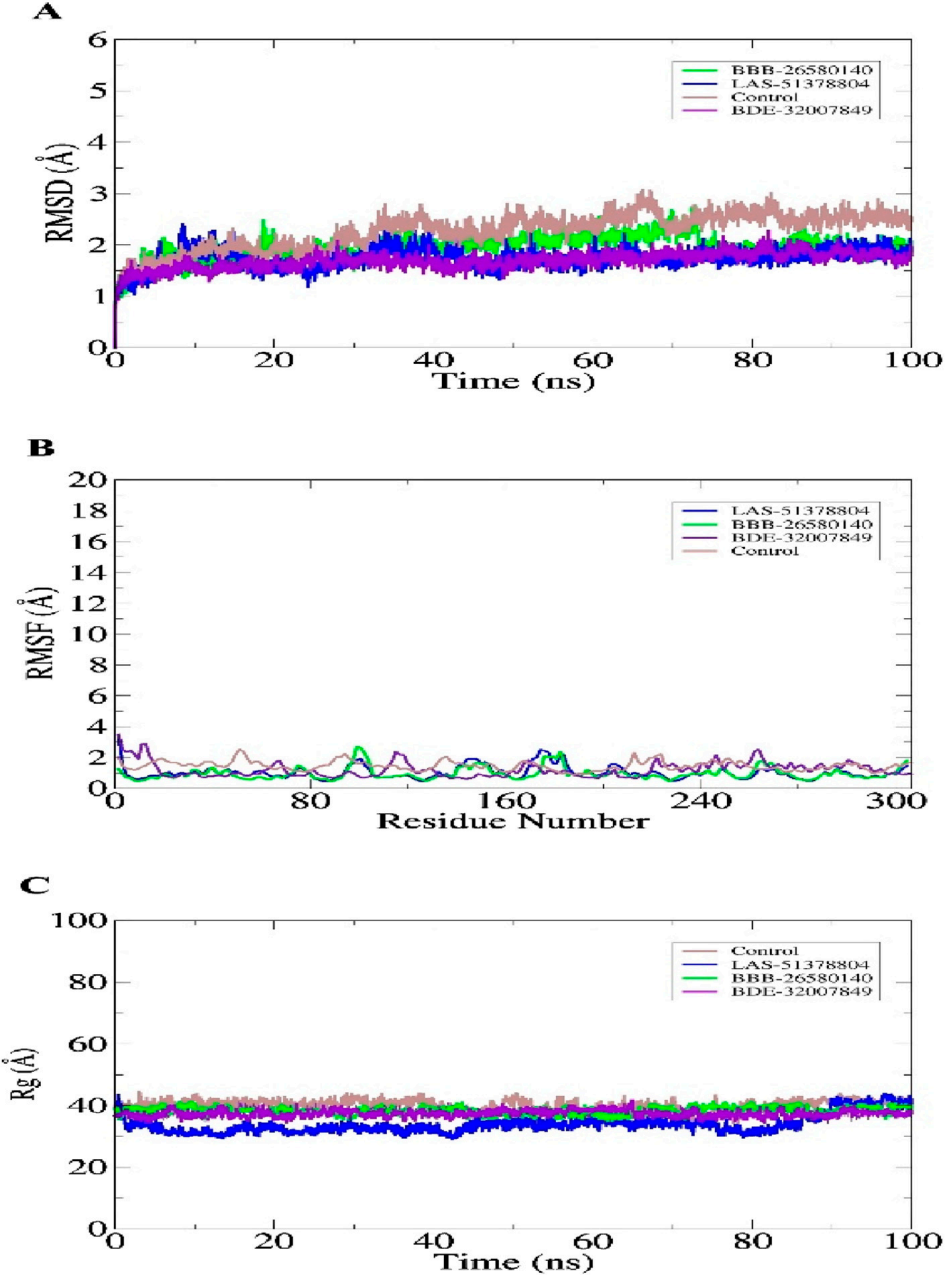
Statistical evaluations performed on the trajectories of the molecular dynamics simulations. The metrics included **(A)** RMSD, **(B)** RMSF, and **(C)** Rg. All units are in angstroms.

Study of the macromolecular structure is a key step in understanding the biology of a molecule. The macromolecules perform their functions by interacting with different ligands. Therefore, stable dynamic behaviors of the docked complexes were interpreted. In molecular dynamics simulations, stable conformations and interactions of the selected leads with the protease enzyme were disclosed. This was essential for the compounds to exert their biological actions by stopping enzyme activity. Statistical analyses were conducted based on different simulations, including the root mean-squared deviation (RMSD) (Ahmad et al., 2017; Maiorov and Crippen, 1994), root mean-squared fluctuation (RMSF) (Ahmad et al., 2017; Kuzmanic and Zagrovic, 2010), and radius of gyration (Rg) (Lobanov et al., 2008). These analyses were conducted by choosing the carbon alpha atoms of the docked systems. The RMSD was performed first; in contrast to the control used, the lead complexes have significantly stable behaviors with no major deviations in the dynamics. The mean RMSDs for BBB-26580140, BDE-32007849, LAS-51378804, and control were 1.53 Å, 1.31 Å, 1.38 Å, and 1.89 Å, respectively (Figure 3A). These values indicate that the control system shows more structural variation in the presence of the control molecule. On the other hand, the lead systems were more tolerant of the presence of the compounds and showed strong intermolecular conformations, making the complexes overall stable in nature. The RMSF values of the complexes were calculated next; the mean RMSF values of BBB-26580140, BDE-32007849, LAS-51378804, and control were 1.35 Å, 1.20 Å, 1.25 Å, and 2.01 Å, respectively (Figure 3B). Here also, larger deviations in the control systems are depicted by the RMSF values; these changes may be attributed to the continuous movements of the control compound in the active pocket of the main protease enzyme, which results in pressure on the flexible regions. Next, the compact nature of the receptor enzyme in the presence of the inhibitors was evaluated. The mean Rg values of BBB-26580140, BDE-32007849, LAS-51378804, and control were 38.52 Å, 37.02 Å, 37.34 Å, and 40.25 Å, respectively (Figure 3C); the Rg values show the same trend as the RMSD and RMSF analyses.

### 3.4 Estimating the binding free energies

The overall objectives of the MM-PBSA and molecular mechanics with generalized Born and surface area (MM-GBSA) are to determine the differences in free energies between the bound and unbound states of the molecules in an aqueous solution and compare different conformations of the molecules (Genheden and Ryde, 2015; Wang et al., 2019). The MM-PBSA and MM-GBSA methods are now regularly applied in drug discovery pipelines as they offer more accurate information than the static intermolecular conformation docking studies (Tuccinardi, 2021). The MM-PBSA requires modest computational resources and is based on diverse molecular dynamics simulation frames (Zhang et al., 2017). The details regarding the MM-PBSA and MM-GBSA binding energies of the complexes are tabulated in Table 2. The values indicate that all three complexes and the control have robust binding energies and that the complexes are considerably stable in terms of the binding and docked conformations. Between the MM-PBSA and MM-GBSA energies, the van der Waals energy demonstrated the most favorable contribution to the equilibrium of the complexes. The net van der Waals energy contributions of BBB-26580140, BDE-32007849, LAS-51378804, and control were found to be −50.36, −52.01, −50.61, and −49.51 kcal/mol, respectively. The electrostatic energy showed the second most favorable contributions to the binding of ligands with the main protease enzyme. The net electrostatic energy contributions to the overall stabilization of the complexes are −16.34, −17.38, −15.00, and −13.69 kcal/mol for BBB-26580140, BDE-32007849, LAS-51378804, and control, respectively. The overall solvation energies of the complexes were found to have non-favorable contributions, and most of these contributions were due to the polar solvation energies. Non-polar solvation energy seems to be vital for stabilization of the complexes. The net solvation energies of BBB-26580140, BDE-32007849, LAS-51378804, and control were found to be 13.68, 12.54, 10.17, and 14.29 kcal/mol for the MM-GBSA as well as 13.10, −57.61, −54.41, and −50.09 kcal/mol for the MM-PBSA, respectively. The overall MM-GBSA binding energies of BBB-26580140, BDE-32007849, LAS-51378804, and control were −53.02, −56.85, −55.44, and −48.91 kcal/mol, respectively. Similarly, the net MM-PBSA binding energies of BBB-26580140, BDE-32007849, LAS-51378804, and control were −53.6, −57.61, −54.41, and −50.09 kcal/mol, respectively.

**TABLE 2:** Free energy estimates of the docked/control complexes. The values are statistically calculated in terms of kcal/mol.

Energy parameter	BBB-26580140	BDE-32007849	LAS-51378804	Control
MM-GBSA				
VDWAALS	−50.36	−52.01	−50.61	−49.51
EEL	−16.34	−17.38	−15.00	−13.69
DELTA G gas	−66.7	−69.39	−65.61	−63.2
DELTA G solv	13.68	12.54	10.17	14.29
DELTA TOTAL	−53.02	−56.85	−55.44	−48.91
MM-PBSA				
VDWAALS	−50.36	−52.01	−50.61	−49.51
EEL	−16.34	−17.38	−15.00	−13.69
DELTA G gas	−66.7	−69.39	−65.61	−63.2
DELTA G solv	13.10	11.78	11.20	13.11
DELTA TOTAL	−53.6	−57.61	−54.41	−50.09

### 3.5 Predictions of the entropy energies of the complexes

**FIGURE 4:**
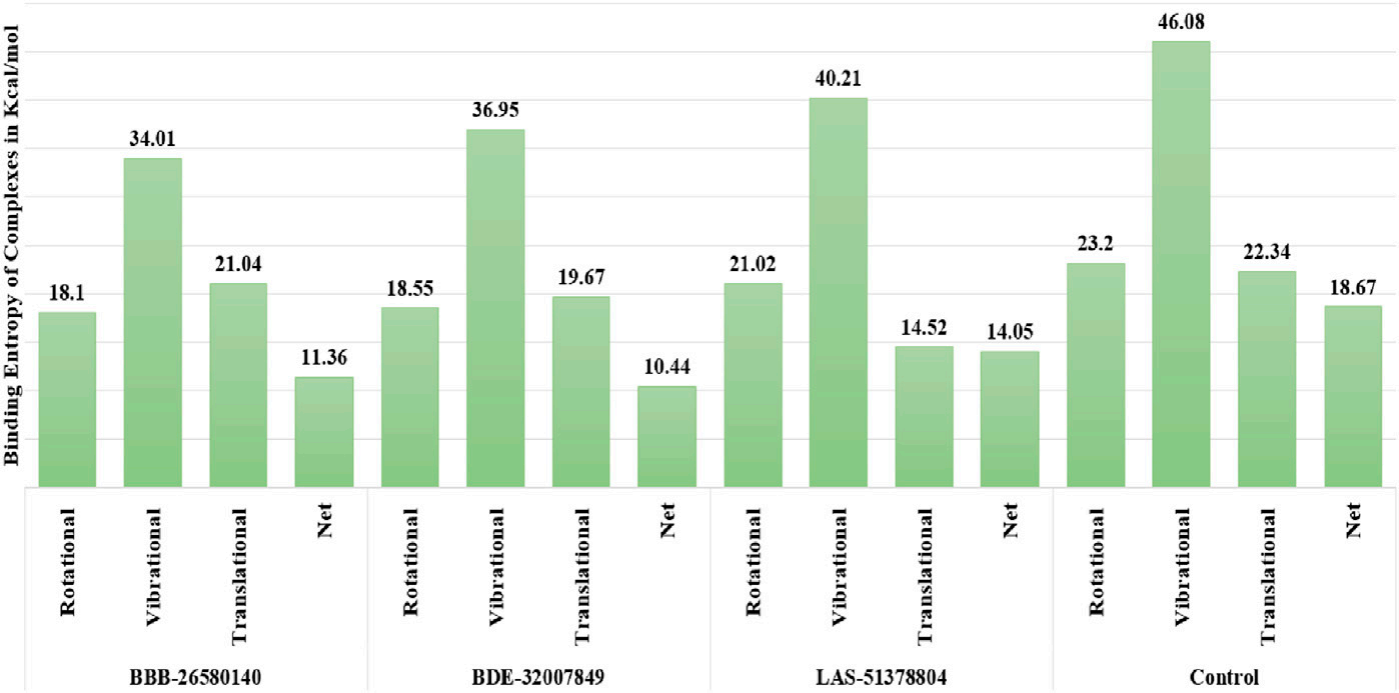
Binding entropy energy contribution of each complex. The total energy is split into the rotational, vibrational, and translational energy components.

The random energy possessed by each complex was determined by entropy analysis. The random free energy enables the ligands to escape the enzyme active pocket and detach from it. It was found that the identified systems had lower free energies. The net entropy energies of BBB-26580140, BDE-32007849, LAS-51378804, and control were 11.36, 10.44, 14.05, and 18.67 kcal/mol, respectively. This additionally confirms that these systems have high intermolecular stabilities and good ligand binding strengths with the protease enzyme. The binding entropy energy findings of the complexes are reported in Figure 4.

### 3.6 Water swap energies of the complexes

**FIGURE 5:**
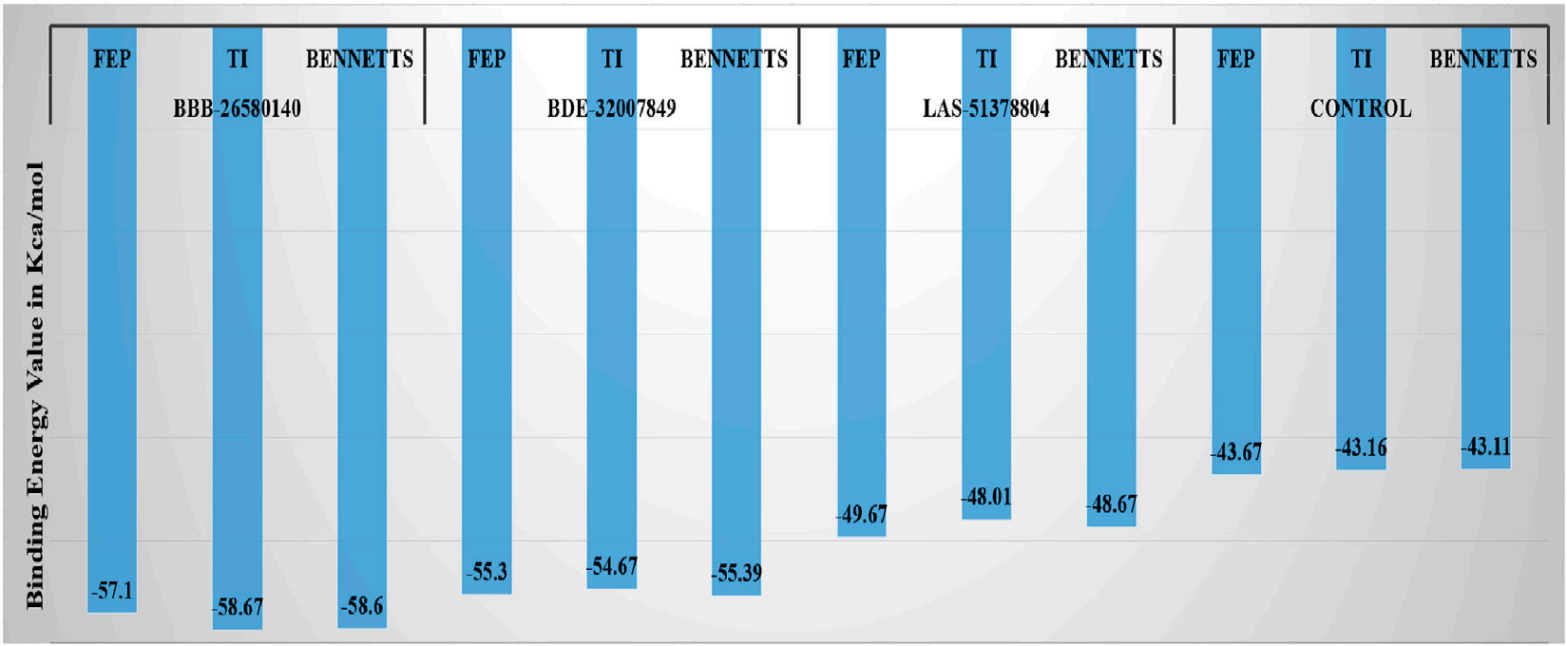
Water-swap-based binding free energies of the complexes in units of kcal/mol.

Next, the water swap binding energies of the complexes were determined to validate and crosscheck the system stabilities. The water swap method is considered to be very robust in terms of confirming the binding affinities of the compounds to the main protease enzyme. Water molecules play significant roles in ligand binding with the receptor enzyme residues (Woods et al., 2014). The water swap approach is particularly useful for investigating the roles of the water molecules in bridging the ligands with the enzyme active site residues (Woods et al., 2011). The water swap method estimates the absolute binding free energy in terms of three algorithms, namely thermodynamic integration (TI), free energy perturbation (FEP), and Bennett’s methods. Among the compounds, BBB-26580140 was found to have the most dominant and stable binding energy. The FEP, TI, and Bennett’s energy values of the compounds were found to be −57.1, −58.67, and −58.6 kcal/mol, respectively. The details of the water swap energies of the complexes are given in Figure 5.

### 3.7 Predictions about drug-likeness and medicinal chemistry of the compounds

Before conducting experimental evaluations, predictions about the drug-likeness of the selected compounds were used to determine whether they could pass the prominent drug-like rules, such as the Lipinski rule of five (Lipinski, 2004), as well as the Veber (Veber et al., 2002), Egan (Egan et al., 2000), and Muegge (Ahmad et al., 2017) rules. Drug-likeness increases the chances that a compound would obtain clearance from the clinical trials for further marketing. Drug-likeness predictions allow us to compare and contrast the selected leads with known drug-like rules. The drug-like molecules also have improved oral bioavailabilities and can be transformed into successful drug molecules (Protti et al., 2021). All three leads and the control molecule were found to show favorable drug-like properties and achieve clearance based on the important drug-like rules, as summarized in Table 3. Similarly, the compounds were found to have good bioavailability scores, meaning that high concentrations of the compounds can reach the target sites for biological actions. The compounds were also disclosed to show no alerts for PAINS, indicating that they may only bind to one specific biological macromolecule and not multiple ones (Whitty, 2011). Thus, there are fewer chances of obtaining false positive results. All the compounds were also found to have good synthetic accessibility scores, where lower scores imply that the compounds can be easily synthesized in the laboratory for biological activities.

**TABLE 3:** Analysis of the medicinal chemistry properties and drug-likeness of the shortlisted inhibitors.

Drug-like rule	BBB-26580140	BDE-32007849	LAS-51378804	Control
Lipinski rule of five	Follow	Follow	Follow	Follow
Egan	Follow	Follow	Follow	Follow
Veber	Follow	Follow	Follow	Follow
Muegge	Follow	Follow	Follow	Follow
Bioavailability score	0.56	0.55	0.56	0.55
Pan-assay interference compounds	No alert	No alert	No alert	No alert
Synthetic accessibility	2.23	3.02	2.26	3.11

### 3.8 Computation of the pharmacokinetic properties

To predict the bodily behaviors upon administration of the selected compounds, the pharmacokinetic properties of the inhibitors were determined (Lombardo et al., 2017). All the lead compounds and the control molecule showed very high gastrointestinal absorption, resulting in the maintenance of significant amounts at the target site for biological activity (Alex, 2003). All compounds were non-permeable to the blood–brain barrier, so the compounds are not expected to have any neurotoxic side effects (De Boer and Breimer, 1994). The compounds were non-substrates of P-glycoprotein, so they will not be expelled from the cells and will be available for biological functions (Wang et al., 2011). The compounds were also found to be non-inhibitors of the cytochrome isoforms (Cheng et al., 2011). Therefore, the compounds can be easily metabolized and excreted from the body after fulfilling their pharmacological actions. Table 4 lists the pharmacokinetic properties of the selected inhibitors and control.

**TABLE 4:** Pharmacokinetic properties of the inhibitory compounds.

Property	BBB-26580140	BDE-32007849	LAS-51378804	Control
Drug absorption form the gastrointestinal tract	Maximum	Maximum	Maximum	Maximum
Penetration of blood–brain barrier	Not detected	Not detected	Not detected	Not detected
Compounds as P-gp substrates	Not detected	Not detected	Not detected	Not detected
Compounds as CYP1A2 receptor inhibitors	Not detected	Not detected	Not detected	Not detected
Compounds as CCYP2C19 receptor inhibitors	Not detected	Not detected	Not detected	Detected
Compounds as CYP2C9 receptor inhibitors	Not detected	Not detected	Not detected	Not detected
Compounds as CYP2D6 receptor inhibitors	Not detected	Not detected	Detected	Not detected
Compounds as CYP3A4 receptor inhibitors	Not detected	Not detected	Not detected	Not detected
Skin permeation (Log *K*_p_)	−7.06 cm/s	−6.49 cm/s	−7.07 cm/s	−7.51 cm/s

### 3.9 Toxicity predictions

The toxicity features of compounds significantly impact their success as drugs. Hence, the toxicities of the selected compounds were evaluated. All compounds were found to be non-toxic, non-immunogenic, and non-mutagenic.

### 3.10 Bioactivity predictions

The possible bioactivities of the leads and control were predicted (Table 5). The molecules with bioactivity scores >0.0 are considered to have considerable biological activities, while those with scores in the range of −0.50 to 0.00 are classified as modestly active and those with values <−0.50 are regarded as inactive (Khan et al., 2017). The findings presented in the table show that the compounds are biologically active and may exert physiological activity by binding to and interacting with the nuclear receptor ligands, protease enzyme, and GPCR ligands. Moreover, the bioactivities of the lead structures were predicted to be more pronounced than that of the control molecule. Among the leads, BDE-32007849 was found to have the most significant biological activities against the target enzymes.

**TABLE 5:** Predicted Mol inspiration bioactivities of the lead/control molecules.

Compound/control	Ligand for GPCR	Modulator of ion channel	Inhibitor of kinase	Ligand for nuclear receptor	Inhibitor of protease	Inhibitor of enzyme
BBB-26580140	0.04	0.09	−0.59	1.01	0.11	0.27
BDE-32007849	0.17	0.14	−0.15	0.15	0.16	0.23
LAS-51378804	0.00	0.09	−0.35	0.66	0.11	0.22
Control	0.13	0.06	−0.22	0.25	−0.005	0.15

## 4 Discussion

The COVID-19 pandemic that started in China and spread globally has resulted in the deaths of millions and left many with health disabilities (Cecconi et al., 2020). Although the disease is currently well-managed with extensive use of effective vaccines, there is an ongoing search for potent drugs (Chukwudozie et al., 2021). In this computational study, diverse drug library sources were screened in an attempt to identify compounds that show the best binding capabilities with the main protease enzyme of SARS-COV-2, the causative agent of the COVID-19 pandemic. As a result, three drug molecules (BBB-26580140, BDE-32007849, and LAS-51378804) were found, which showed highly stable conformations with the enzyme at the S1 pocket. The docking binding energy scores of BBB-26580140, BDE-32007849, and LAS-51378804 were −13.02, −13.0, and −12.56 kcal/mol, respectively. These energy scores determine the compounds as lead structures with networks of strong intermolecular interactions. In contrast to the control molecule Z1741970824 (binding energy score: −11.59 kcal/mol), the three compounds showed robust hydrophilic and hydrophobic interactions. The interactions most important to the overall stabilities of the complexes involve the enzyme active site residues, such as His41 and Cys145. As the molecular-docking-based virtual screening often results in false positive findings, the predicted stabilities of the best molecular bindings with the main protease enzyme of SARS-CoV-2 were validated through molecular dynamics simulations. It was observed that the enzyme structures were stable in the presence of the shortlisted leads and that the networks of binding interactions were intact. The net MM-GBSA binding energies of BBB-26580140, BDE-32007849, LAS-51378804, and control were −53.02, −56.85, −55.44, and −48.91 kcal/mol, respectively; the net MM-PBSA binding energies of BBB-26580140, BDE-32007849, LAS-51378804, and control were −53.6, −57.61, −54.41, and −50.09 kcal/mol, respectively. These energies demonstrate the formation of strong intermolecular complexes. Moreover, the predicted binding entropy energies of the systems showed the presence of lower free energies, indicating that the systems had stable nature. From the drug-likeness perspective, the compounds were found to be non-toxic while fulfilling the important drug-like rules. The compounds also revealed good ADMET properties (Jia et al., 2019). Thus, the predicted compounds are promising lead structures that can be utilized in further *in vitro* and *in vivo* studies.

## 5 Conclusion

In this study, three drug molecules (BBB-26580140, BDE-32007849, and LAS-51378804) were identified as promising binders of the main protease enzyme of SARS-CoV-2 after extensive structure-based virtual screening of four libraries (Asinex Antiviral, Seaweed Metabolite Database, MFSMTL, and CMNPD with 6,827, 1,191, 1,830, and 45,000 compounds, respectively). All three compounds showed highly stable binding conformations at the S1 active pocket of the main protease enzyme and formed short-distance hydrophilic and hydrophobic contacts with catalytically active residues. The stable intermolecular conformations of the docked complexes were validated through molecular dynamics simulations, which showed minor local loop-mediated deviations and the absence of global changes. It was also noted that the enzyme was tolerant to the presence of the compounds when they occupied the active pocket. The compounds also revealed stable electrostatic and van der Waals energies as well as lower polar solvation energies. Additionally, the compounds were proven to be drug-like and showed favorable medicinal chemistry properties along with non-toxic, non-mutagenic, and non-carcinogenic features. The compounds were found to show bioactivities against nuclear receptor ligands, protease enzymes, GPCRs, etc. The bottom line of this study is that the identified compounds are promising and may be used in further experimental tests to validate their real biological potentials.

## Data Availability

The original contributions presented in the study are included in the article/supplementary material; further inquiries can be directed to the corresponding author.
